# The *Arabidopsis* small G‐protein AtRAN1 is a positive regulator in chitin‐induced stomatal closure and disease resistance

**DOI:** 10.1111/mpp.13010

**Published:** 2020-11-15

**Authors:** Zhiqiang Song, Cheng Zhang, Ling Chen, Pinyuan Jin, Charles Tetteh, Xiuhong Zhou, Zhimou Gao, Huajian Zhang

**Affiliations:** ^1^ Department of Plant Pathology College of Plant Protection Anhui Agricultural University, Key Laboratory of Biology and Sustainable Management of Plant Diseases and Pests of Anhui Higher Education Institutes Hefei Anhui China

**Keywords:** chitin, elicitor, plant defence, signaling pathways, small G‐protein, stomatal closure

## Abstract

Chitin, a fungal microbial‐associated molecular pattern, triggers various defence responses in several plant systems. Although it induces stomatal closure, the molecular mechanisms of its interactions with guard cell signalling pathways are unclear. Based on screening of public microarray data obtained from the ATH1 Affymetrix and Arabidopsis eFP browser, we isolated a cDNA encoding a Ras‐related nuclear protein 1 AtRAN1. *AtRAN1* expression was enriched in guard cells in a manner consistent with involvement in the control of the stomatal movement. *AtRAN1* mutation impaired chitin‐induced stomatal closure and accumulation of reactive oxygen species and nitric oxide in guard cells. In addition, *Atran1* mutant plants exhibited compromised chitin‐enhanced plant resistance to both bacterial and fungal pathogens due to changes in defence‐related genes. Furthermore, *Atran1* mutant plants were hypersensitive to drought stress compared to Col‐0 plants, and had lower levels of stress‐responsive genes. These data demonstrate a previously uncharacterized signalling role for AtRAN1, mediating chitin‐induced signalling.

## Graphical abstract

Guard cell‐expressed *Arabidopsis* AtRAN1 mediates chitin‐induced stomatal closure and disease resistance through regulating reactive oxygen species, nitric oxide production, and defence‐related gene expression.

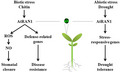

## 1 INTRODUCTION

Plants coexist with diverse microorganisms, and their interactions have resulted in coevolution. Higher plants have immune systems with two different levels to resist pathogen invasion and multiplication. The gene‐for‐gene concept characterizes genetic control by plant resistance gene products and pathogen avirulence proteins, which are pathogen‐secreted effectors that manipulate host processes in favour of the pathogen (Goehre & Robatzek, 2008; Gouveia et al., 2017; Wang et al., 2019); their direct or indirect molecular interactions initiate effector‐triggered immunity (ETI), which is essential to conventional race‐specific resistance breeding programmes (Peng et al., 2018). Moreover, microbial‐originated invariant structures, known as microbial‐associated molecular patterns (MAMPs), are able to activate pattern‐triggered immunity (PTI) in various cultivars of numerous species, resulting in the elicitation of plant stomatal closure and disease resistance (Boller & He, 2009; Chisholm et al., 2006).

Chitin is a polymer of *N*‐acetyl‐d‐glucosamine that can be obtained from natural sources, such as arthropod exoskeletons or fungal cell walls. It is a PTI inducer that elicits stomatal closure and disease resistance, and has been applied to control crop disease (Liu et al., 2019; Miya et al., 2007; Wan et al., 2008). Recognition of chitin by rice requires chitin elicitor binding protein (OsCEBiP), which contains an extracellular lysine motif but lacks an intracellular kinase domain (Akamatsu et al., 2013). An additional receptor, OsCERK1, interacts with OsCEBiP to form a heterodimer that transduces extracellular signals within cells (Shimizu et al., 2010). OsRacGEF1 and OsRLCK185 are direct substrates of OsCERK1 for transducing chitin signals (Akamatsu et al., 2013; Yamaguchi et al., 2013). In *Arabidopsis*, the cell surface receptor AtCERK1 binds to chitin, leading to homodimerzation of the receptor and subsequent activation of innate immunity (Liu et al., 2012; Wan et al., 2008). In addition to AtCERK1, the receptors AtLYK4 and AtLYK5 can bind to chitin and are essential to the chitin response (Wan et al., 2012). AtLYK5 has a higher affinity for chitin than AtCERK1, and is probably the primary receptor for chitin recognition (Cao et al., 2014). Although studies have been conducted to elucidate chitin signal transduction in induced disease resistance, many components of the transduction pathway, particularly those related to chitin‐activated stomatal closure, remain unknown.

Stomata, which are natural openings in the plant surface bordered by guard cells, provide portals for pathogen penetration. In particular, foliar bacterial pathogens reach the interior of the plant mainly through stomata to cause disease. Active control of stomatal closure hampers pathogen invasion (Sawinski et al., 2013). When an *Arabidopsis* leaf is exposed to fungi or chitin, its guard cells respond by closing the stomatal aperture, hampering pathogen penetration (Lee et al., 1999). Chitin‐induced stomatal closure is dependent on intracellular signalling via reactive oxygen species (ROS) and nitric oxide (NO), as demonstrated by pharmacological assays (Khokon et al., 2010). AtCERK1 is expressed in guard cells and is required for *Fusarium oxysporum‐*triggered nonhost resistance and stomatal closure in *Arabidopsis* (Huang et al., 2017; Khokon et al., 2010; Liu et al., 2009). Furthermore, AtCERK1‐PBL27 (receptor‐like cytoplasmic kinase, RLCK)‐SLAH3 (anion channel protein) constitutes a short signal transduction module that regulates chitin‐induced stomatal closure and antifungal immunity (Liu et al., 2019). Characterization of more proteins involved in the guard cell and plant resistance signal transduction pathways will help to clarify the relationship between stomatal movement and disease resistance.

Stomatal movements are regulated by abiotic and biotic stresses, including light, drought, CO_2_, humidity, microbes, plant hormones, and elicitors (Murata et al., 2015). The mechanisms underlying regulation of stomatal movement involve receptors, protein kinases, ion channels, and second messengers such as ROS and NO, indicating that stomatal guard cells of the plants harbour dynamic regulatory networks in plants (Shi et al., 2015). Molecular factors related to guard cell signalling induced by chitin could be exploited in the future as switches to activate resistance in the design of efficient strategies to protect crops.

By high‐throughput down‐regulation screening of a plant cDNA library, we identified several genes driving stomatal closure or plant cell death in response to elicitors, including *NbVPE*, *NbGa*, *NbMAPKKKa*, *NbMEK2*, *NbWIPK*, and *NbALY916* (Teng et al., 2014; Zhang et al., 2010, 2012a, 2012b). The evolutionarily conserved small‐G protein RANs have emerged as key signalling proteins regulating multiple cellular processes in animals and yeast, including nuclear translocation of proteins and RNA, cell cycle regulation, and nuclear envelope maintenance (Reiner & Lundquist, 2018). RANs associated with guanine nucleotide exchange factors (GEFs) and GTPase‐activation proteins (GAPs) act as molecular switches that are activated by GTP and inactivated by the hydrolysis of GTP to GDP (Vernoud et al., 2003). In animals, exportin‐1 binds RAN and nuclear export signal (NES)‐containing proteins to form a complex used for nuclear export and antiviral immunity (Heaton et al., 2019; Li et al., 2019). In plants, RAN can interact with AtXPOI, an *Arabidopsis* exportin‐1 homologue, and AtRanBP1, an NES‐containing protein, in biochemical assays (Haasen et al., 1999). RANs have been found to play roles in seed development, vegetative growth, and abiotic stress responses (Liu et al., 2014; Xu & Cai, 2014; Xu et al., 2016), but their biological function has not been fully elucidated. Here, we found that Ras‐related nuclear protein 1 (AtRAN1), which is a small G‐protein, is highly expressed in *Arabidopsis* guard cells. *Atran1* mutant plants exhibited impaired in chitin‐induced stomatal closure, resistance to bacterial and fungal pathogens, and drought tolerance. Our results suggest a role in chitin signalling for a small G‐protein in *Arabidopsis*.

## 2 RESULTS

### 2.1 Structure of AtRAN1

**FIGURE 1:**
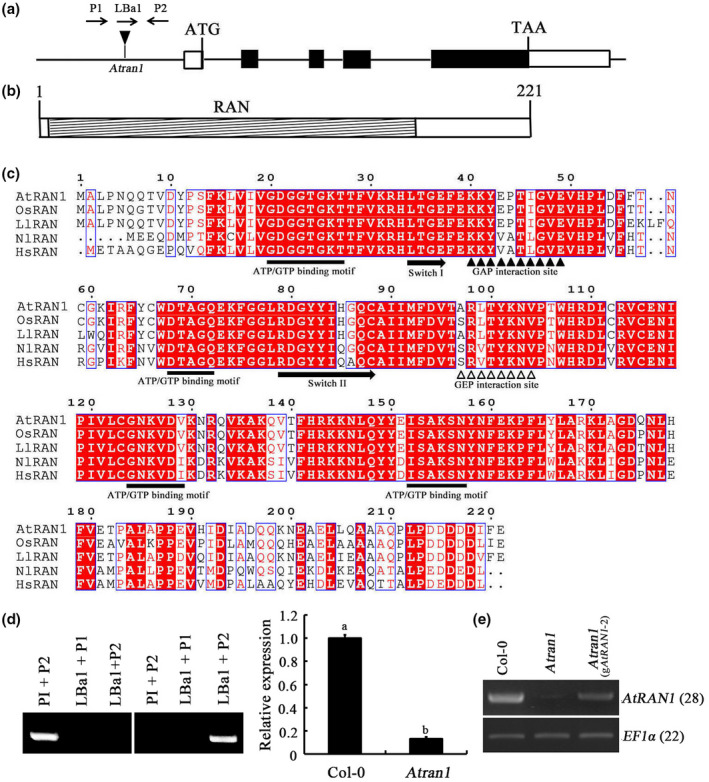
Structure of the*AtRAN1*gene and AtRAN1. (a) Structure of*AtRAN1*. Black boxes, coding sequence; white boxes, 5′ (80 nt) and 3′ (347 nt) untranslated regions; horizontal line, intron sequence. (b) Structure of AtRAN1. Hatched box, RAN domain (amino acids 7–171). (c) Sequence alignment and structures of RANs from*Arabidopsis*(AED92779.1),*Nilaparvata lugens*(KT313028.1),*Lepidium latifolium*(GU014818.1),*Homo sapiens*(BC051908.2), and*Oryza sativa*(AB015287.1). Filled rectangles, domain elements. Amino acids with 100% conservation are shaded red. Gaps were introduced to permit alignment. (d) PCR verification of the*Atran1*T‐DNA mutant and quantitative reverse transcription PCR (RT‐qPCR) analysis of the homozygous T‐DNA insertion line to determine the level of*AtRAN1*transcript relative to Col‐0 plants.*S16*and*EF1α*were used as internal controls. (e) Recovery of*AtRAN1*expression in the*Atran1*mutants complemented with wild‐type*AtRAN1*gene.*AtRAN1*transcript levels were determined by semiquantitative RT‐PCR

*AtRAN1* contained five exons and four introns, a 5′ untranslated region of 80 nucleotides (nt), and a 3′ untranslated region of 347 nt (Figure 1a). *AtRAN1* is predicted to encode a protein of 221 amino acids containing a RAN domain protein (Figure 1b). AtRAN1 contained four ATP/GTP‐binding motifs, two effector molecule‐binding motifs (GAP and GEP interaction sites), and two switch regions (Switch I and II). GTPase‐activating protein and guanine‐nucleotide‐exchange factor interaction sites (GEFs) were located at positions 44–49 and 97–104, respectively, which are conserved among various plant RANs. The highly acidic C‐terminal motif DDDDDIFE was present at positions 214–219, although it differed slightly from other plant and insect RANs (Figure 1c).

**FIGURE 2:**
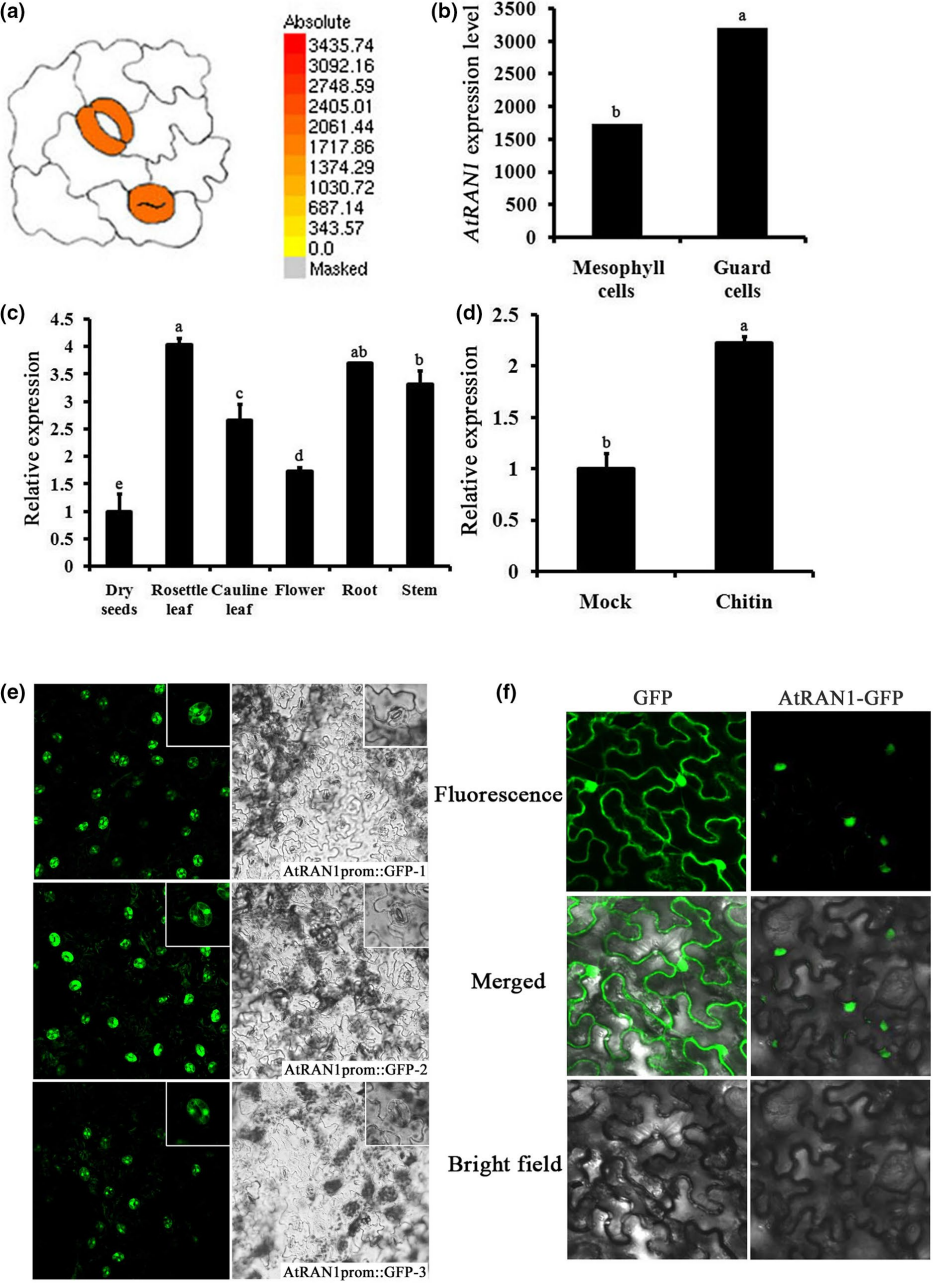
*AtRAN1*expression in plants and AtRAN1 subcellular localization. (a) The expression of*AtRAN1*in guard cells was monitored by microarray data from ATH1 Affymetrix (MIAMExpress, accession number: E‐MEXP‐1443; Yang et al.,^2008^) and*Arabidopsis*eFP browser (http://www.bar.utoronto.ca/efp/cgi‐bin/efpWeb.cgi; Winter et al.,^2007^). (b) Gene Chip Operating Software‐normalized expression levels of*AtRAN1*in leaf cells and guard cells were retrieved using*Arabidopsis*eFP Browser. (c) Quantitative reverse transcription PCR (RT‐qPCR) analysis of the*AtRAN1*expression in dry seeds, rosette leaf, cauline leaf, flower, root, and stem. (d) RT‐qPCR analysis of*AtRAN1*expression upon chitin treatment. In all cases,*AtRAN1*transcript levels were normalized to*S16*and*EF1α*levels. Error bars indicate*SE*. Each bar represents an average of three independent reactions, including both biological and technical replicates. (e) Visualization of*AtRAN1*promoter::green fluorescent protein (GFP) expression in the epidermis of transgenic*Arabidopsis*lines (*AtRAN1*promoter::GFP‐1, 2, 3) by confocal microscopy.(f) Transient expression of AtRAN1‐GFP fusion protein in*Nicotiana benthamiana*leaves. AtRAN1‐GFP expression was driven by cauliflower mosaic virus 35S promoter and transiently expressed in*N. benthamiana*leaves by agroinfiltration

Previous studies have shown that chitin triggers stomatal closure. *Arabidopsis AtRAN1* is ubiquitously expressed during plant development, regulating seed development as well as abiotic stress tolerance (Haizel et al., 1997; Liu et al., 2014, 2019), and raising the possibility that AtRAN1 may be involved in the response to chitin. To study the potential role of *AtRAN1* in chitin‐induced stomatal closure, in silico analysis of *AtRAN1* expression in *Arabidopsis* guard cells was performed with publicly available expression data using the ATH1 Affymetrix protocal (MIAMExpress, accession number: E‐MEXP‐1443, Yang et al., 2008) and *Arabidopsis* eFP browser (http://www.bar.utoronto.ca/efp/cgi‐bin/efpWeb.cgi; Winter et al., 2007), which contains microarray databases for guard cells and other tissues. *AtRAN1* had a high expression level in guard cells (Figure 2a). As expected from transcriptomic data, *AtRAN1* mRNA was detected through quantitative reverse transcription PCR (RT‐qPCR) in all tissues tested, and was up‐regulated upon chitin treatment (Figure 2b,c,d).

### 2.2 *AtRAN1*prom::GFP activity is observed in stomatal guard cells

We created *AtRAN1* promoter/green fluorescent protein (*AtRAN1*prom::GFP) reporter lines and randomly selected transgenic lines to examine *AtRAN1* promoter activity in stomata and stomatal precursor cells. Analyses of multiple independent transgenic lines expressing the *AtRAN1*prom::GFP construct showed GFP activity in the guard cells of developing true leaves. *AtRAN1* was expressed in guard cells of the abaxial leaf surface throughout the lifetime of the plant under normal conditions. The GFP signals were observed preferentially in nuclei and secondarily in the cytosol of guard cells (Figure 2e). The obvious localization of AtRAN1 in guard cells suggests it to be a regulator of stomatal function. Therefore, *AtRAN1* promoter activity is associated with stomata.

To further explore the subcellular localization of AtRAN1, an AtRAN1‐GFP fusion protein driven by the cauliflower mosaic virus 35S promoter was transiently expressed in *Nicotiana benthamiana* leaf cells. *Agrobacterium*‐mediated transient transformation of *N. benthamiana* leaves has emerged as the method of choice for achieving high levels of in planta transgene expression within a week, which makes it a powerful system for testing subcellular distribution (Rolland, 2018). Confocal imaging indicated that the fluorescence signal was primarily confined to the nucleus, with a weak signal in the cytoplasm, whereas the control GFP was distributed throughout the cytoplasm and nucleus at 36 hr postinfiltration. Thus, AtRAN1 was mainly localized to the nucleus (Figure 2f). This finding is consistent with those from the rice and mammalian counterparts of AtRAN1 (Quimby & Dasso, 2003; Xu et al., 2016).

### 2.3 Chitin‐induced stomatal closure is compromised in *Atran1* mutant plants

The increase in *AtRAN1* expression in response to chitin prompted us to assess the role of AtRAN1 in chitin signalling. We employed a homozygous line containing T‐DNA insertions of *AtRAN1* from the SALK T‐DNA insertion collection. The *Atran1* mutants were verified through diagnostic PCR screening and DNA sequencing, and contained a T‐DNA insertion 492 bp upstream of the ATG start codon. Using RT‐qPCR, we verified a large reduction in *AtRAN1* transcript levels (Figure 1d). Two rounds of backcrossing were performed to reduce possible genetic background effects. *Atran1* mutants had no apparent phenotypic differences compared to Col‐0 plants under physiological growth conditions.

**FIGURE 3:**
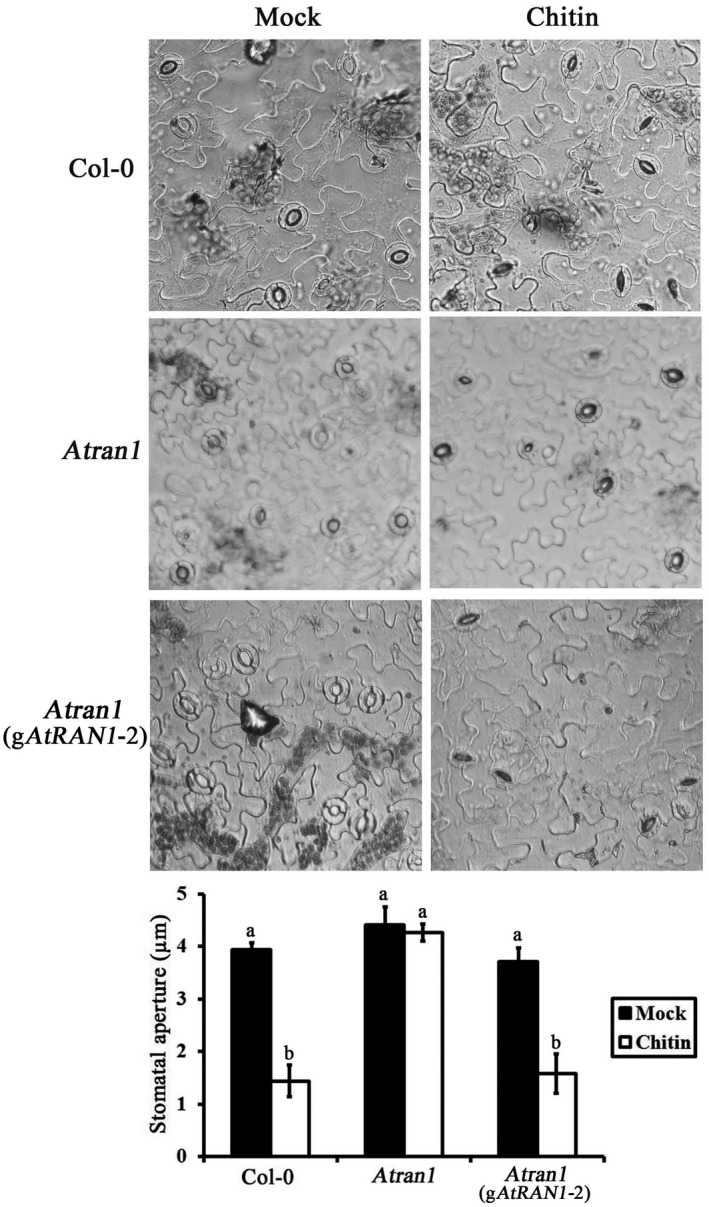
Sensitivity of the*Atran1*mutant to chitin treatment. Leaves of Col‐0,*Atran1*, and g*AtRAN1*plants with open stomata were exposed to either mock or 50 µg/ml chitin. Representative images from one of these experiments are shown. The stomatal apertures were measured after a 2‐hr incubation. At least 60 stomata were measured for each genotype per replication. Data were means ± *SE*of three independent experiments

Various biotic and abiotic stresses can induce stomatal closure (Vahisalu et al., 2008). Given that *AtRAN1* was up‐regulated by chitin in *Arabidopsis* leaves, we investigated the responses of *Atran1* mutant guard cells to chitin. Col‐0 plants exhibited stomatal closure upon chitin treatment; however, no significant response of the *Atran1* mutant was observed in response to chitin. Moreover, relative to Col‐0 plants, *Atran1* mutant plants had larger stomatal apertures upon chitin treatment (Figure 3). To verify that these changes occurred as direct responses to chitin, we used an *Atcerk1* mutant (chitin receptor) as a positive control. As expected, the *Atcerk1* mutant showed no stomatal closure upon chitin treatment, exhibiting a visible stoma phenotype (Figure S1), as reported previously (Liu et al., 2019). To determine whether the reduction in sensitivity to chitin‐induced stomatal closure is attributable to disruption of the *AtRAN1* gene, we introduced a wild‐type copy of the *AtRAN1* gene into the *Atran1* mutant. Transformants were identified through hygromycin selection and *AtRAN1* expression was confirmed through RT‐PCR (Figure 1e). Examination of five randomly chosen, independent T_1_ g*AtRAN1* lines showed similar stomatal apertures compared to Col‐0 plants upon chitin treatment. We selected a transgenic line (#2) for further analyses. Thus, mutation of *AtRAN1* hampered the chitin‐induced stomatal closure.

### 2.4 AtRAN1 acts upstream of ROS and NO production to generate chitin‐induced stomatal closure

**FIGURE 4:**
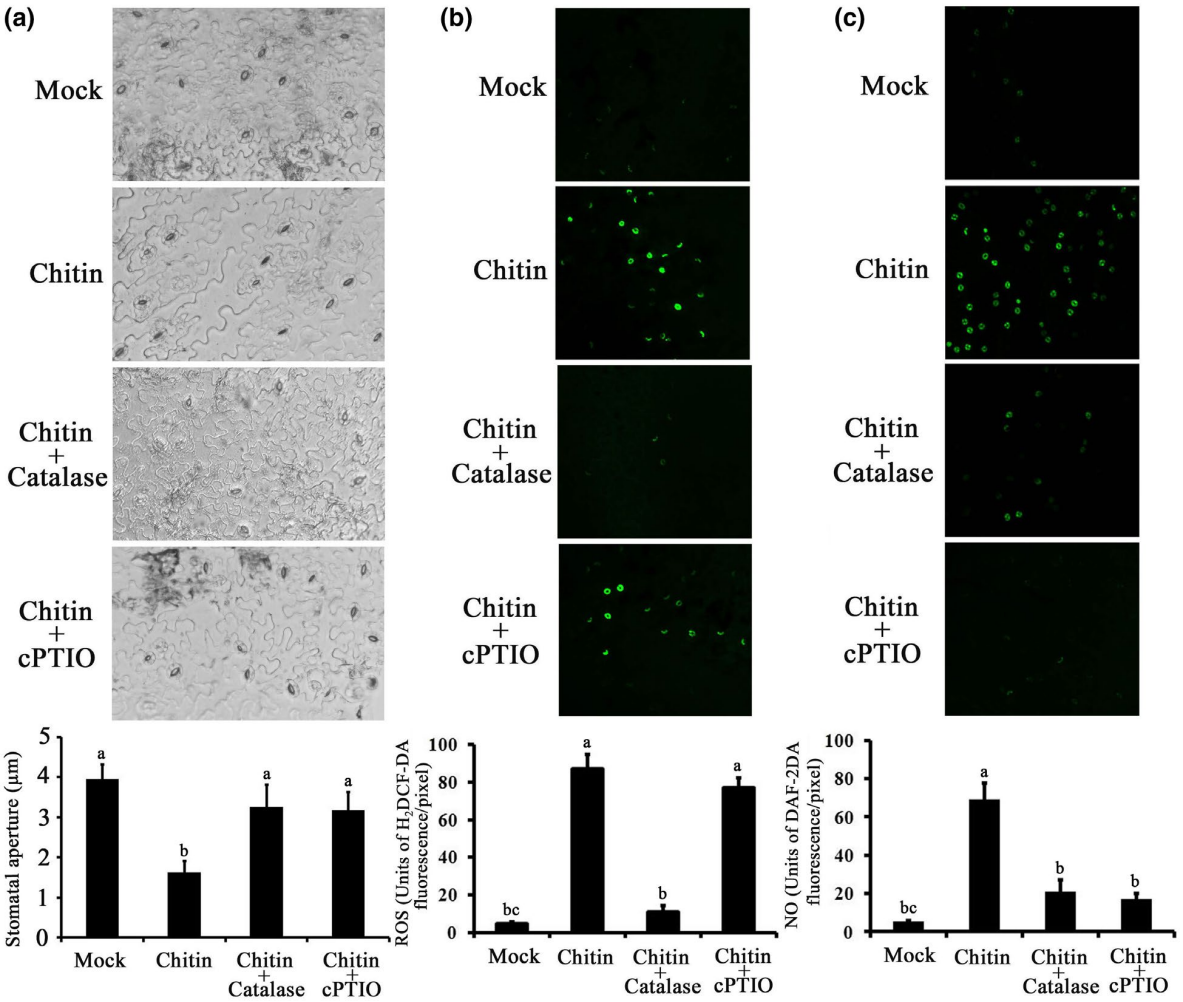
Involvement of reactive oxygen species (ROS) and nitric oxide (NO) in chitin signalling in*Arabidopsis*guard cells. (a) Effects of 100 units/ml catalase and 100 µM cPTIO on 50 µg/ml chitin‐induced stomatal closure. After treatment, stomatal apertures were observed in epidermal fragments from abaxial surfaces of the treated leaves. Values represent means ± *SE*from three independent experiments;*n* = 50 apertures per experiment. (b) Effects of catalase and cPTIO on chitin‐induced ROS production in guard cells. Fluorescence images and pixel intensities in guard cells preloaded with 50 µM H_2_DCF‐DA for 20 min in darkness were recorded. (c) Effects of catalase and cPTIO on chitin‐induced NO production in guard cells. Fluorescence images and pixel intensities in guard cells preloaded with 20 µM DAF‐2DA for 20 min in darkness were recorded. Data of fluorescence pixel intensities are displayed as means ± *SE*,*n* = 50 apertures per experiment. Means with different letters denote statistically significant differences among different treatments as determined by analysis of variance (LSD test,*p* < .05)

Both ROS and NO are essential second messengers that function in guard cell signalling. We examined the effects of a ROS scavenger (catalase) and a NO scavenger (cPTIO) on the chitin‐induced stomatal closure (Figure 4a). Chitin‐induced stomatal closure was significantly inhibited by exogenous application of catalase and cPTIO, whereas catalase or cPTIO alone did not induce stomatal movement (data not shown). We further monitored ROS and NO synthesis in guard cells using the ROS‐ and NO‐sensitive fluorescent dyes H_2_DCF‐DA and DAF‐2DA, respectively, by confocal microscopy. As shown in Figure 4, chitin affected both ROS and NO production, with obvious fluorescence observed in guard cells. Catalase sharply reduced chitin‐induced ROS production, whereas cPTIO had no effect on chitin‐induced ROS production (Figure 4b). Similarly, chitin‐triggered NO accumulation was markedly inhibited by catalase and cPTIO (Figure 4c). As expected, exogenously applied hydrogen peroxide (H_2_O_2_, a major type of ROS) enhanced NO fluorescence in guard cells (Figure S2), which is consistent with previous reports (Xie et al., 2014). Together, the correlation between stomatal movement and changes in ROS and NO levels indicate that ROS mediate chitin‐induced stomatal closure via synthesis of NO.

**FIGURE 5:**
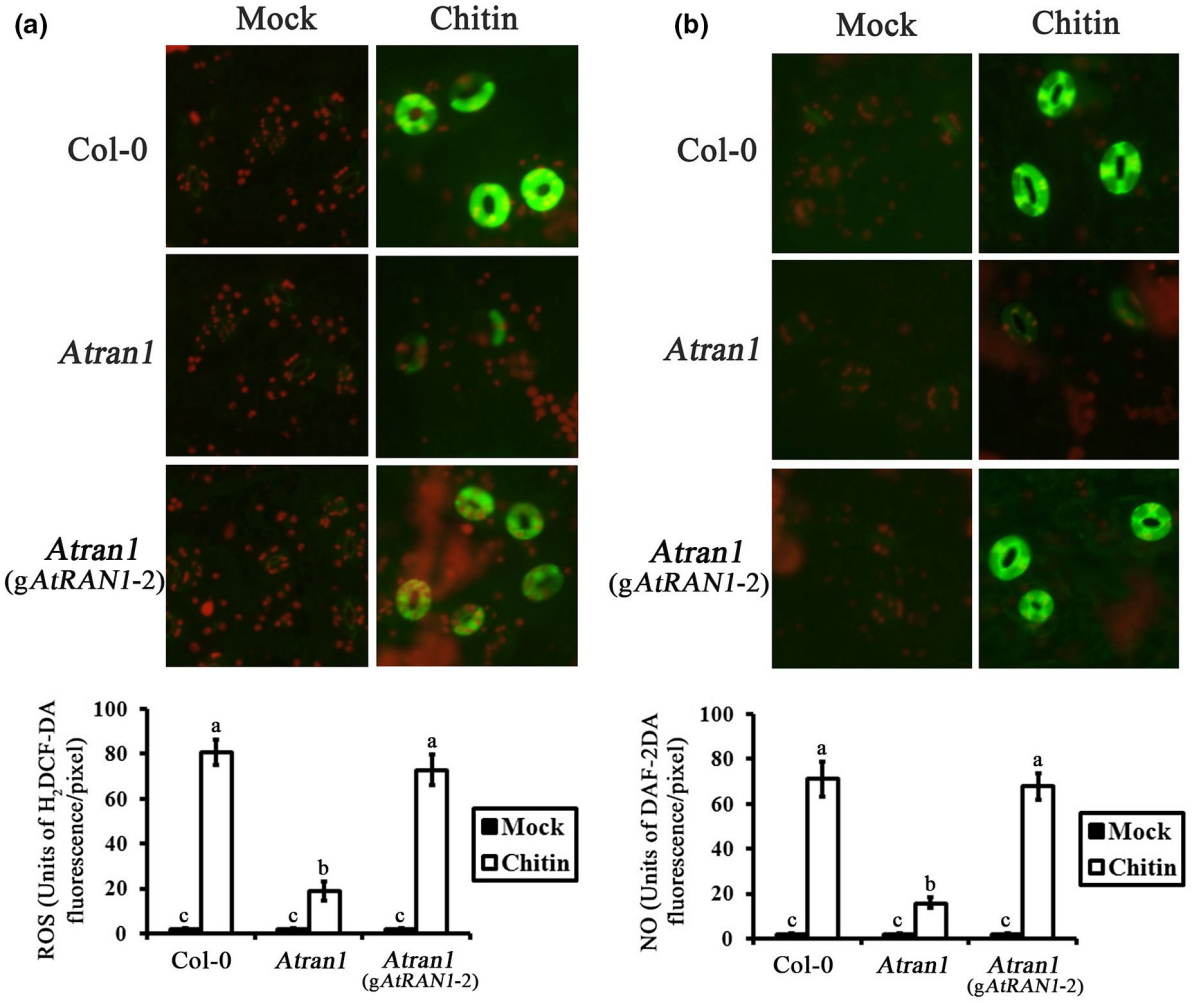
Reactive oxygen species (ROS) and nitric oxide (NO) levels in stomata of*Atran1*mutant plants after chitin treatment. (a) H_2_DCF‐DA was loaded into the epidermal peels cells, and fluorescence images and pixel intensities were recorded in the guard cells of Col‐0,*Atran1*mutant, and g *AtRAN1*plants after addition of 50 µg/ml chitin. (b) DAF‐2DA was loaded into epidermal peels cells, and fluorescence images and pixel intensities were recorded in guard cells of Col‐0,*Atran1*mutant, and g*AtRAN1*plants after addition of 50 µg/ml chitin. Each measurement is the average of three replicates; experiments were repeated twice with similar results. Data for fluorescence intensity of ROS and NO generation are presented as means ± *SE*of three independent experiments

Next, we investigated the relationships among AtRAN1, ROS, and NO in the guard‐cell chitin‐signalling network. We observed obvious ROS fluorescence in Col‐0 guard cells after chitin treatment. Only low‐intensity ROS fluorescence was observed after mock treatment. Fluorescence intensity in *Atran1* mutant guard cells was reduced after chitin treatment. No differences in ROS fluorescence intensity were observed in g*AtRAN1* lines guard cells compared to Col‐0 plants in response to chitin (Figure 5a).

We conducted similar analyses of NO accumulation in *Atran1* mutant plants. Figure 5b shows chitin‐induced NO accumulation in Col‐0 guard cells. *Atran1* mutant plants showed inhibition of the effects of chitin and blocked stomatal closure, as well as abolished NO production. Guard cells of g*AtRAN1* lines showed similar NO production compared to Col‐0 plants upon chitin treatment. These data suggest that AtRAN1 mediates chitin‐induced stomatal closure by regulating ROS and NO production.

### 2.5 *AtRAN1* loss‐of‐function affects chitin‐induced disease resistance to bacterial and fungal pathogens

**FIGURE 6:**
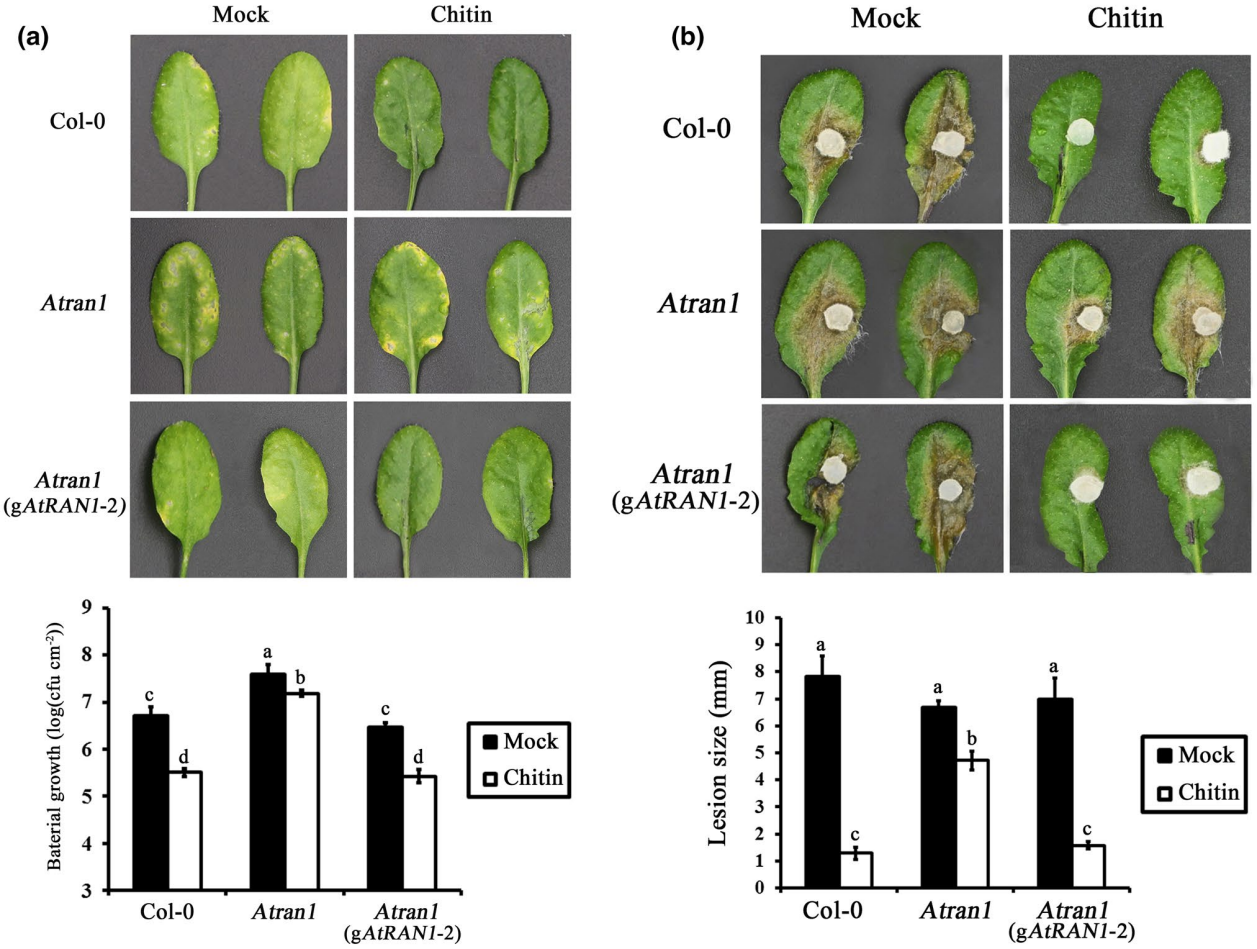
The resistance of*Atran1*mutant plants to*Pseudomonas syringae*pv.*tomato*(Pst) DC3000 and*Sclerotinia sclerotiorum*in response to chitin. (a) Disease symptoms caused by Pst DC3000 on individual leaves of indicated*Arabidopsis*lines. Plants were photographed 5 days postinoculation and 1 day after chitin treatment. Representative two leaves from one of three experiments are shown. Quantitative analysis of bacterial growth in planta after inoculation with Pst DC3000. (b) Five‐week‐old mature plants were pretreated (24 hr before pathogen inoculation) with chitin, and then, leaves were inoculated with *S. sclerotiorum* by attaching a 4 mm diameter hyphal plug onto surface of detached leaves. Representative two leaves from one of three experiments are shown. Lesion size was measured 24 hr after inoculation. Means ± *SE* of three independent experiments are shown. Statistical analysis was carried out using a two‐way analysis of variance (ANOVA) with GraphPad Prism software. Different letters denote statistically significant differences among different treatments as determined by ANOVA (LSD test,*p* < .05)

To further investigate the roles of chitin signalling and AtRAN1 in plant defence, both Col‐0 and *Atran1* mutant plants were pretreated with chitin and their responses to different pathogens were examined. We tested the effects of chitin pretreatment on infection by *Pseudomonas syringae* pv. *tomato* (Pst) DC3000 of Col‐0 and *Atran1* mutant plants. One day after chitin treatment, plants were dip‐inoculated with 10^8^ cfu/ml of Pst DC3000. Mock‐treated *Atran1* mutant plants were more susceptible than Col‐0 to Pst DC3000, as indicated by a 7.62‐fold greater number of bacteria present 4 days after inoculation as well as increased chlorotic spots on day 5 (Figure 6a). In addition, bacterial abundances in chitin‐treated *Atran1* mutant plants were reduced by 2.59‐fold compared to those in mock‐treated *Atran1* plants, reflecting the general induction of plant innate immunity in both Col‐0 and *Atran1* mutant plants in the presence of chitin. g*AtRAN1* lines and Col‐0 plants displayed similar disease symptoms and bacterial titres of Pst DC3000 with chitin treatment. However, the chitin‐induced disease resistance of the *Atran1* mutant was of lower magnitude than that in Col‐0 plants. Thus, AtRAN1 may be involved in chitin‐induced resistance to Pst DC3000.

To test the specificity of AtRAN1 in chitin‐induced bacterial disease resistance, the response of the *Atran1* mutant to the fungal pathogen *Sclerotinia sclerotiorum* upon chitin treatment was also examined (Figure 6b). Col‐0 plants pretreated with chitin exhibited less severe disease symptoms upon inoculation with *S. sclerotiorum*, as reflected by the reduced lesion size. However, such pretreatment of the *Atran1* mutant plants did not enhance resistance. These data further support the critical role of AtRAN1 in chitin signalling for fungal resistance.

### 2.6 Loss of function of *AtRAN1* affects the transcription of defence‐related genes

**FIGURE 7:**
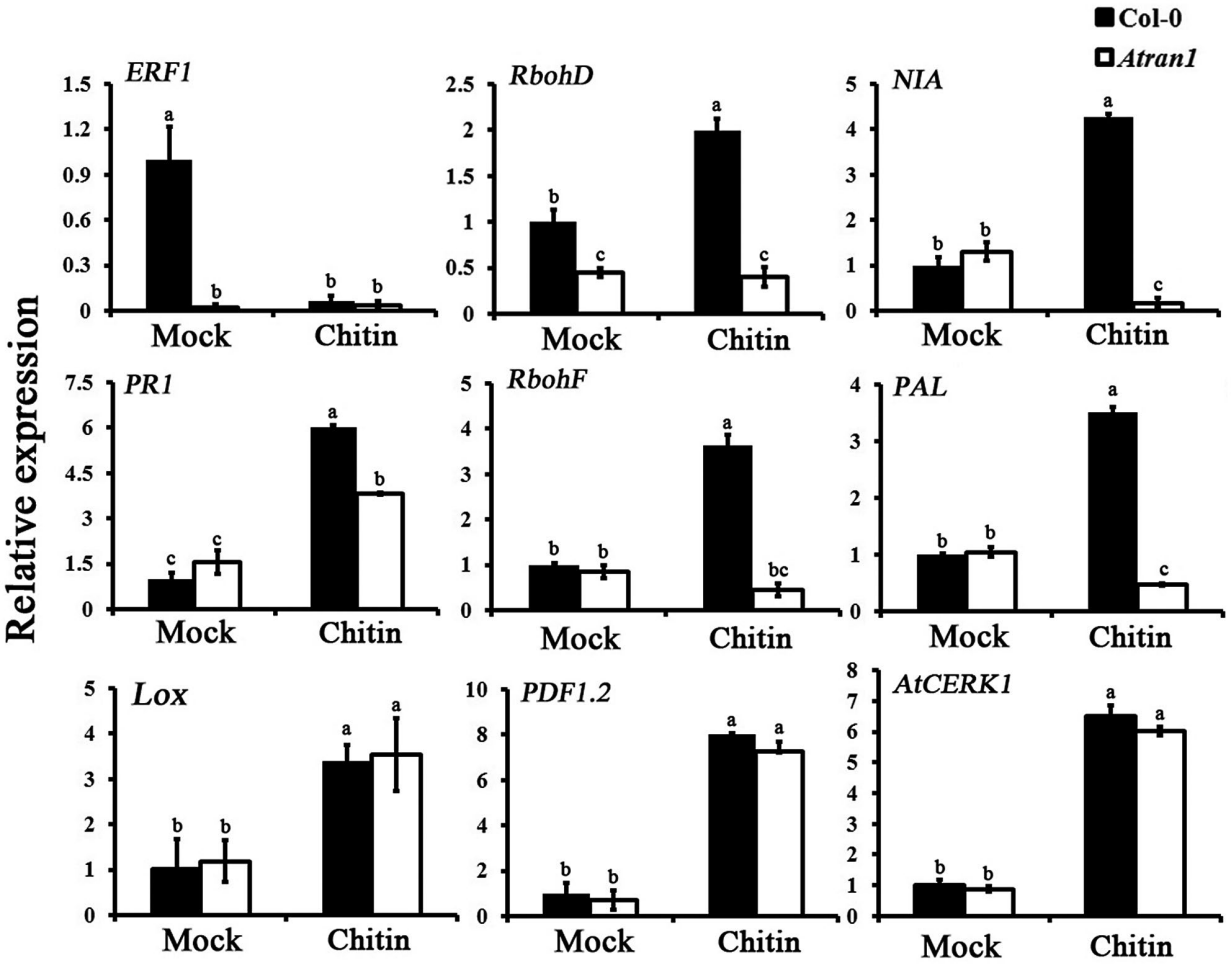
Expression analyses of the indicated defence‐responsive genes in*Atran1*mutant plants in response to chitin. Samples (*n* = 5) with or without chitin treatment were collected. Values represent means ± *SE*from three independent experiments; different letters denote statistically significant differences among different treatments as determined by ANOVA (LSD test,*p* < .05)

Compromised disease resistance in *Atran1* mutant plants after chitin treatment may affect the expression of defence‐related genes. To address this possibility, the transcript levels of nine selected genes, including those known to function in the salicylic acid (SA)‐, jasmonic acid (JA)‐, and chitin plant defence‐signalling pathways, were examined in *Atran1* mutant and Col‐0 plants. The expression of nine genes was influenced by chitin treatment in four expression patterns (Figure 7). In the *Atran1* mutant plants, expression of the *ethylene responsive factor 1* (*ERF1*) gene was reduced, with mRNA levels at 3% or less of those in mock‐treated Col‐0 plants, and did not significantly differ after chitin treatment. The expression of the *respiratory burst oxidase homolog D* (*RbohD*) gene was 2‐fold and 5‐fold lower in mock‐ and chitin‐treated *Atran1* mutant plants, respectively, than in Col‐0 plants. Compared to Col‐0 plants, the transcript levels of the *nitrate reductase* (*NIA*), *acidic pathogenesis‐related 1* (*PR1*), *respiratory burst oxidase homolog F* (*RbohF*), and *phenylalanine ammonia‐lyase* (*PAL*) genes did not significantly differ in mock‐treated *Atran1* mutant and Col‐0 plants, but were 1.5‐fold to 25‐fold lower in chitin‐treated *Atran1* mutant than in Col‐0 plants. The expression levels of *lipoxygenase* (*Lox*), *plant defensin 1.2* (*PDF1.2*), and *chitin receptor* (*AtCERK1*) genes did not markedly differ between mock‐ and chitin‐treated *Atran1* mutant or Col‐0 plants. Compared to mock treatment, the expression of *NIA* and *PAL* were down‐regulated in the *Atran1* mutant in response to chitin. The AtRAN1‐mediated inhibition effect on gene expression is probably dependent on time after chitin treatment. Some chitin‐responsive genes, including *RbohD*, *NIA*, *PR1*, *RbohF*, and *PAL*, were affected in *Atran1* mutant plants, while others, including *ERF1*, *Lox*, *PDF1.2*, and *AtCERK1*, were not. Together, the data suggest that AtRAN1 may contribute to chitin‐induced plant resistance to bacterial and fungal pathogens through effects on certain defence‐related genes.

### 2.7 *Atran1* mutant plants show reduced tolerance to drought stress

**FIGURE 8:**
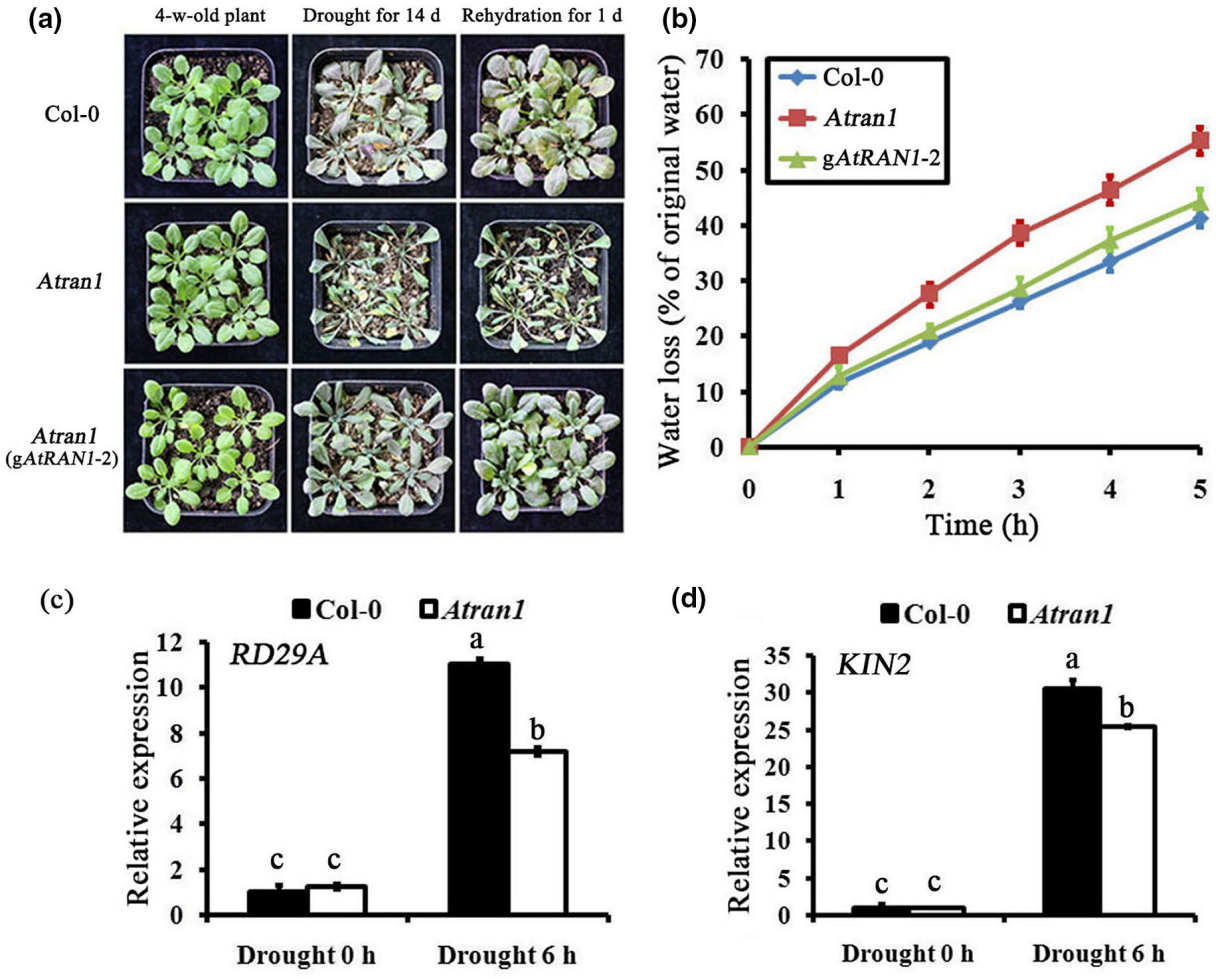
Sensitivity of the*Atran1*mutant to drought. (a) Appearance of Col‐0,*Atran1*mutant, and g*AtRAN1*plants grown under drought stress. The experiments were repeated three times with similar results. (b) Water‐loss assay in Col‐0,*Atran1*mutant and g*AtRAN1*plants. Four or five leaves of 5‐week‐old plants were cut from the base and subjected to water‐loss assay. Data are means ± *SE*of three replicates, each evaluating 15 plants. (c) and (d) The transcription of the indicated stress‐responsive genes was analysed in*Atran1*mutant plants by exposure to drought stress for 6 hr

A higher expression level of AtRAN1 in guard cells was related to chitin‐induced stomatal closure and disease resistance, suggesting positive regulation of AtRAN1 in biotic stress. Drought is the most destructive abiotic stress affecting plant transpiration and causing noninfectious plant disease. Therefore, we investigated whether AtRAN1 modulates plant tolerance to drought stress. *Atran1* mutant and Col‐0 plants were grown with sufficient water for approximately 4 weeks, and all plants appeared relatively health with similar phenotypes; subsequently, water was withheld for 14 days. The *Atran1* mutant leaves showed serious wilting and the plants became moribund, whereas 100% of Col‐0 plants survived (Figure 8a). The plants were rewatered and their phenotypes recorded. The *Atran1* mutant was relatively sensitive to drought. Moreover, detached leaves of *Atran1* mutant plants lost water more rapidly than those of Col‐0 plants. g*AtRAN1* lines showed a similar level of water loss to that of Col‐0 plants (Figure 8b). RT‐qPCR profiling showed that the transcript levels of drought stress‐responsive genes (*RD29A* and *KIN2*) did not differ before drought stress, but were 1.2‐fold to 1.5‐fold lower in response to drought in *Atran1* mutant plants than in Col‐0 plants (Figure 8).

## 3 DISCUSSION

The observations described here convincingly show that *Arabidopsis* AtRAN1 is highly expressed in guard cells and plays essential roles in chitin‐responsive control of stomatal closure, activation of ROS or NO production, and disease resistance, as well as drought stress tolerance. Therefore, we suggest that AtRAN1 could be explored for improving plant tolerance to abiotic and biotic stresses.

RAN is an evolutionarily conserved eukaryotic GTPase. RAN is considered an essential factor for nuclear transportation of protein in animal cells and plays a role in animal immunity against virus infection (Han & Zhang, 2007). Recent studies have demonstrated that plant RAN might participate in cell cycle control, postmitotic nuclear assembly, auxin sensitivity, and response to environmental stress (Chen et al., 2011; Wang et al., 2006; Zang et al., 2010). The results presented here support the pivotal roles of *Arabidopsis* RAN in stomatal movement, disease resistance, and the drought stress response. These results are consistent with previous findings that *Arabidopsis* and rice plants overexpressing RAN show improved cold tolerance (Xu & Cai, 2014; Xu et al., 2016), and provide compelling evidence that these RANs are essential to the abiotic stress response, revealing both conserved and plant‐specific roles in accordance with their counterparts in animals.

In this study, AtRAN1 was expressed in guard cells throughout the lifespan of the plants. The *Atran1* mutant exhibited altered responses to chitin‐induced stomatal closure and drought, indicating that guard cell localization of AtRAN1 may be associated with stomatal movement. Recent data have demonstrated AtCERK1‐PBL27‐SLAC3 is a signal transduction module and is responsible for chitin‐induced stomatal closure and antifungal immunity (Liu et al., 2019). In rice, OsCERK1‐dependent chitin signalling involves both RLCKs and OsRacGEFs. OsCERK1‐OsRacGEF1‐OsRac1‐MKK4‐OsMAPK3/6 and OsCERK1‐RLCK185‐OsMAPK3/6 are two proposed pathways activated upon chitin reception (Akamatsu et al., 2013; Kim et al., 2012; Wang et al., 2017). As AtRAN1 contains two typical GEFs that control the activities of RAN GTPases (Figure 2c) and AtRANGEFs have the capacity for GEF activity, further identification of AtRANGEFs that activate AtRAN1 and kinases that function downstream of AtRAN1 will help to clarify chitin signalling in *Arabidopsis*. We found that *Arabidopsis* AtRAN1, similar to AtCERK1, can regulate chitin‐induced stomatal closure, as well as plant resistance. *Atran1* mutation did not affect chitin‐induced *AtCERK1* expression, and that interaction analyses for AtRAN1 with AtCERK1 and other chitin signalling components should be conducted. As similar chitin‐recognition patterns mediated by CERK1 are found in *Arabidopsis* and rice, whether *Arabidopsis* AtRAN1 employs a similar mechanism to that of rice OsRac1 in response to chitin remains to be investigated.

Studies of several crop species, including rice, tomato, and *Arabidopsis*, showed that proteins can regulate elicitor‐triggered stomatal movement and plant resistance (Du et al., 2014; Qiu & Yu, 2009). The tomato NAC transcription factor JA2 is highly expressed in guard cells of tomato leaves. *JA2*‐silenced tomato plants show impaired Pst DC3000‐ and flg22‐induced stomatal closure (Du et al., 2014). Furthermore, *JA2*‐silenced plants contain greater pathogen biomass than wild‐type plants during pathogen assays (Du et al., 2014). In our study, AtRAN1 is a guard‐cell expressed protein and stomatal closure was less sensitive to chitin treatment in the *Atran1* mutant than in Col‐0 plants. Consistent with a positive role in chitin‐triggered stomatal closure, *Atran1* mutant plants contained more Pst DC3000 biomass than wild‐type plants upon chitin treatment. This result may be interpreted as due to compromised stomatal closure in *Atran1* mutant plants, which provides a portal through which more bacteria move into the stoma and get into leaf apoplast to cause disease. As chitin is an important component of the fungal cell wall, enhanced susceptibility to *S. sclerotiorum* upon mutation of *AtRAN1* is unsurprising. These data raise the possibility that AtRAN1 may also be involved in the response to bacterial pathogen‐associated molecular patterns (PAMPs), although this hypothesis remains untested. In addition, *Atran1* mutant plants showed reduced tolerance to drought stress, suggesting AtRAN1 plays dual roles in biotic and abiotic signalling, and is involved in the crosstalk between signalling pathways activated by abiotic and biotic stresses. In a recent research study, the plant drought stress is influenced by the hormone abscisic acid (ABA) (Yan et al., 2020), and thus further investigation of ABA signalling in relation to AtRAN1‐mediated chitin signalling in the context of stomatal closure or drought stress will help to identify the common and unique processes in the response to biotic and abiotic stresses. Plants are simultaneously subjected to a variety of stresses. Understanding the crosstalk in converging stress response pathways may be helpful for increasing crop stress tolerance and crop production.

ROS and NO are strongly associated with plant defence responses. In this study, the ROS and NO levels in guard cells were lower in the *Atran1* mutant than in Col‐0 plants after chitin treatment. Several groups have reported that *Arabidopsis RbohD* and *RbohF* play roles in ROS generation and modulate the response to bacterial and oomycete pathogens (Torres et al., 2002, 2017; Trujillo et al., 2006). Our results showed that the expression pattern of *RbohD* was similar in *Atran1* mutant and Col‐0 plants with and without chitin treatment, while *RbohF* expression was down‐regulated in *Atran1* mutant upon chitin treatment, suggesting involvement of *Rboh* in an *AtRAN1*‐dependent pathway related to chitin‐induced ROS accumulation. NIA is important in the biosynthesis of NO, and *Arabidopsis NIA*‐dependent NO production is key to the establishment of resistance to disease caused by Pst DC3000 (Lozano‐Juste & Leon, 2010; Vitor et al., 2013). The expression level of *NIA* was reduced in the *Atran1* mutant after chitin treatment. This result suggests that chitin‐induced AtRAN1‐mediated stomatal closure and resistance to pathogens may be related to *Rboh*‐dependent ROS accumulation and *NIA*‐dependent NO accumulation. Our previous work and this study indicate that elicitors with different origins can induce ROS and NO production (Zhang et al., 2010, 2014, 2016a, 2016b). Therefore, these results indicate that intracellular ROS and NO accumulation is shared among all signalling pathways in guard cells, and signalling components such as AtRAN1 mediate the intracellular accumulation of ROS and NO in guard cells. Such pathways may interconnect, with specificity of plant signal transduction achieved through transcriptional changes in response to different pathogen elicitors. Previous studies have reported the collinearity of chitin, ROS, and NO in guard cell signalling (Khokon et al., 2010). Pharmacological evidence indicates that ROS‐induced NO generation in guard cells is related to both nitric oxide synthase and nitrate reductase activity (Khokon et al., 2010). This finding suggests further complexity of chitin‐guard cell signalling, which has not yet been fully described. Collectively, these data suggest a role for AtRAN1 upstream of ROS and NO production in chitin‐mediated stomatal closure signalling.

Mutation of *AtRAN1* affected susceptibility to the plant hemibiotrophic pathogen Pst DC3000 and necrotrophic pathogen *S. sclerotiorum* with and without chitin treatment. These results suggest that *AtRAN1* mutation impairs either basic defence or chitin‐induced defence in plants. The expression of defence‐related genes showed that *AtRAN1* mutation decreases *ERF1* expression, suggesting that *AtRAN1* may regulate JA signalling and thereby affect the basic plant defence response. AtRAN1‐mediated defence induced by chitin is probably related to the effects of AtRAN1 on SA signalling, and AtRAN1 positively regulates the expression of chitin‐responsive genes involved in SA signalling. Mutation of *AtRAN1* decreases the chitin‐induced expression of SA marker genes (Kim & Hwang, 2014) such as *PR1* and *PAL*. In contrast, the expression of JA and ethylene (ET) marker genes, such as *ERF1*, *PDF1.2*, and *Lox*, was similar in *Atran1* mutant and Col‐0 plants in response to chitin. Although most studies have reported an antagonistic interaction between the SA and JA/ET signalling pathways, positive crosstalk also occurs (Clarke et al., 2000; Robert‐Seilaniantz et al., 2011). In addition, *AtRAN1* mutation did not affect *AtCERK1* expression upon chitin treatment, suggesting that AtRAN1 may act downstream of AtCERK1 or in an AtCERK1‐independent manner in chitin signalling. Thus, AtRAN1 may function as a positive regulator of SA signalling pathways related to chitin‐triggered plant defences. Further investigations of the roles of AtRAN1 in disease resistance to various biotic stresses, such as bacterial flg22 and fungal chitin, will help to reveal the relationships among AtRAN1‐mediated stomatal movement, stomatal immunity, and apoplast immunity in plants.

Here, we identified and characterized AtRAN1 as a guard cell‐localized small G‐protein that responds to chitin and drought. *AtRAN1* mutation impaired chitin‐induced stomatal closure, leading to lower ROS and NO accumulation in guard cells. Moreover, *AtRAN1* mutation compromised chitin‐induced plant resistance to Pst DC3000 and *S. sclerotiorum*, while also decreasing expression levels of defence‐related genes. In addition, AtRAN1 may be a positive regulator of the drought response via pathways mediated by stress responsive genes. Follow‐up research will aim to identify the targets of AtRAN1 to further elucidate the signalling pathways used by plants exposed to abiotic and biotic stresses.

## 4 EXPERIMENTAL PROCEDURES

### 4.1 Plant materials and growth conditions

Col‐0 and *Atran1* mutant (SALK_067649) were obtained from the Arabidopsis Biological Resource Center (Rhee et al., 2003). Seeds were germinated on half‐strength Murashige and Skoog (0.5 × MS) medium containing 1% wt/vol sucrose and 0.8% (wt/vol) agar for 7–8 days. Next, seedlings were transplanted into soil and grown for a further 4–5 weeks under a photoperiod of 12 hr (150 μmol⋅m^−2^⋅s^−1^), a constant temperature of 22 °C, and a constant relative humidity of 70%.

### 4.2 Chemicals

4,5‐diaminofluorescein‐2 diacetate (DAF‐2DA), 2′,7′‐dichlorodihydrofluorescein diacetate (H_2_DCFDA), catalase, diphenyleneiodonium chloride (DPI), salicylhydroamic acid (SHAM), and 2‐(4‐carboxyphenyl)‐4,4,5,5‐tetramethylimidazoline‐1‐oxyl‐3‐oxide (cPTIO) were obtained from Sigma‐Aldrich. The remaining chemicals of the highest analytical grade were bought from Sangon Biotech.

Chitin oligosaccharide containing 80% chitooctaose (octa‐*N*‐acetyl), 1% chitohexaose, 10% chitoheptaose, and 9% chitononaose was obtained from Dalian GlycoBio.

### 4.3 Bioinformatics analysis of AtRAN1

The entire *AtRAN1* open reading frame (ORF) was amplified from *Arabidopsis* cDNA by RT‐PCR using primers (AtRAN1‐F and AtRAN1‐R), according to AGI ID At5g20010. The PCR product was gel purified, ligated into pMD19 (TaKaRa), and transformed into *Escherichia coli* DH5α competent cells. Recombinant plasmids were confirmed by sequencing. The *AtRAN1* sequence was characterized by identifying ORFs using the National Center for Biotechnology Information Open Reading Frame Finder (ORF Finder) (http://www.ncbi.nlm.nih.gov/gorf/gorf.html). The 5′ and 3′ untranslated regions were retrieved using *Arabidopsis* TAIR Browser. The molecular formula and molecular weight of AtRAN1 protein were predicted and calculated using ExPASy. Domain analysis was performed using InterProScan sequence search (http://www.ebi.ac.uk/interpro/search/sequencesearch?t = 1,407,759,093,265). Amino‐acid sequences of AtRAN1 homologues from other organisms were obtained from the NCBI website. The *Lepidium latifolium* sequence originated from update OCT 21, 2009 NCBI annotation. The *Homo sapiens* sequence was obtained from HUGO Nomenclature Committee annotation. Other sequences included the annotation release 101 from *Oryza sativa* and Release 100 from *Nilaparvata lugens*. Multiple alignments were created using ClustalW2 software.

Arabidopsis eFP Browser (http://bar.utoronto.ca/efp_arabidopsis/cgi‐bin/efpWeb.cgi) is a web‐based tool that can help to explore large‐scale microarray data to permit intuitive visualization of gene expression data across approximately 22,000 genes from *Arabidopsis thaliana* (Winter et al., 2007). First, choose the data source of tissue specific from the Arabidopsis eFP Browser, then fill the AGI ID (Arabidopsis Genome Initiative identifier) of candidate genes. Upon submission in the “Absolute” mode, the plant tissues are coloured ranging from yellow to red, according to the expression level of the gene of interest. The genes with high expression level in guard cells were selected to screen the most reliable reference genes for chitin‐triggered expressions via RT‐qPCR.

### 4.4 Plasmid construction and *Arabidopsis* transformation

The AtRAN1 promoter was cloned by PCR using gene‐specific primers (AtRAN1promoter‐F and AtRAN1promoter‐R) that amplified nucleotides −612 to +6, and the resulting AtRAN1 promoter fragment containing all the required *cis*‐regulatory elements was directly cloned into the pBIGFP (S65T) binary vector (Kang et al., 2008) using the *Sal*I and *Bam*HI unique restriction sites.

To make transient expression construct pBinGFP2::AtRAN1, the *AtRAN1* gene was cloned using cDNA from *Arabidopsis* with primers AtRAN1‐TL‐F and AtRAN1‐TL‐R. The PCR products were inserted into *Kpn*I‐ and *Sal*I‐digested pBinGFP2, creating a fusion with the GFP coding region.

For the molecular complementation experiment, a 2.202‐kb fragment that included a 557‐bp region upstream of the start codon and a 261‐bp region downstream of the stop codon of *AtRAN1* was amplified by PCR from genomic DNA using the primers AtRAN1‐ResF and AtRAN1‐ResR, which introduced *Kpn*I and *Bam*HI sites at each end, respectively. The resulting PCR product was cloned into pCAMBIA1300 (CAMBIA) using the *Kpn*I and *Bam*HI sites, producing plasmid pCAMBIA1300::gAtRAN1. Constructs were verified by sequencing and used to transform Col‐0 and *Arabidopsis Atran1* mutant plants using a modified floral dip procedure (Clough & Bent, 1998).

### 4.5 Analysis of GFP expression

Tissues from plants containing GFP constructs were analysed using an FV1000 confocal laser scanning microscope (Olympus) to determine GFP expression in guard cells. The abaxial epidermal strips from independent seedlings leaves were peeled gently, mounted on a microscope slide, and examined with the microscope, with an argon laser at a wavelength of 488 nm and emission between 500 and 530 nm, as described by Zhang et al. (2018) and Liang et al. (2005), and they displayed coherent results.

For transient expression of the GFP fusion proteins, constructs expressing pBin‐AtRAN1‐GFP and vector alone (pBin‐GFP) were transformed into *A. tumefaciens* GV3101. For infiltration, recombinant strains were cultured in Luria‐Bertani medium supplemented with 50 mg/ml kanamycin in a test tube at 28 °C and 220 rpm for 48 hr. The cells were collected by centrifugation, washed, and then resuspended in MMA buffer (10 mM MgCl_2_, 1 mM 2‐(*N*‐morpholino) ethanesulfonic, pH 5.6, 100 μM acetosyringone) to an optical density at 600 nm of 1.0. Infiltration experiments were performed on 7‐ to 8‐week‐old *N. benthamiana* plants. After 36 hr, confocal laser scanning microscopy with excitation at 488 nm and emission at 500–530 nm was carried out to visualize subcellular localization of AtRAN1‐GFP.

### 4.6 Pathogen assays

Pst DC3000 was provided by Barbara Kunkel (Washington University, USA). Bacteria were cultivated at 28 °C and 220 rpm in King's B medium containing 50 mg/ml rifampicin. Pst DC3000 inoculation assays were performed as described previously (Yao et al., 2013). A bacterial suspension (10^8^ cfu/ml) was dip‐inoculated onto rosette leaves of 5‐week‐old *A. thaliana*. Leaf discs collected 4 days postinoculation (dpi) were washed twice with sterile water and homogenized in 10 mM MgSO_4_. Bacteria were enumerated at 4 dpi as described by Yang et al. (2015). Each biological repeat comprised nine leaf discs from three separate plants.

*S. sclerotiorum* NGA4 was grown on potato dextrose agar as described in the method of Zhang et al. (2014). Leaves were detached from 5‐week‐old *A. thaliana* and transferred onto filter paper saturated with sterile distilled water in a Petri dish. A 4‐mm diameter hyphal plug was then placed upside down onto each leaf and kept under a 16 hr day/8 hr night regime at 25 °C. Pictures of the lesions were taken at 1 dpi, and lesion diameters were recorded.

### 4.7 Stomatal bioassays

Stomatal closure bioassays were performed as described previously (Zhang et al., 2016b). Two whole leaves were excised per genotype and immediately transferred to dishes containing MES‐KCl buffer (10 mM MES‐KOH, 50 mM KCl, pH 6.15), incubated under light for 3 hr to open stomata. Once the stomata were fully open, epidermal strips were peeled off and treated with various inhibitors for a further 30 min under light conditions with or without 50 µg/ml chitin treatment. The abaxial epidermis was photographed at 400× magnification. Stomatal apertures were measured using digital photographs and Fluoview viewer software.

### 4.8 Fluorescence imaging of ROS and NO production

ROS and NO were assayed using the fluorescent indicator dyes H_2_DCF‐DA and DAF‐2DA, respectively, as described previously (Khokon et al., 2011) with minor modifications. Leaves were incubated in MES‐KCl buffer for 3 hr under white light (150 µmol⋅m^−2^⋅s^−1^). The incubated leaves were sliced into <3 mm^2^ pieces, which were then incubated for 20 min in darkness with 50 µM H_2_DCF‐DA. After 50 µg/ml chitin treatment, guard‐cell pairs were imaged with an Olympus FV1000 confocal laser scanning microscope or fluorescence microscopy with excitation at 488 nm and emission at 530 nm. Images were processed using Photoshop software and the average fluorescence intensities in guard cells were analysed. For NO detection, 20 µM DAF‐2DA was added instead of 50 µM H_2_DCF‐DA.

### 4.9 RT‐qPCR analysis

Total RNAs were extracted from seedlings using TRIzol (Invitrogen) according to the manufacturer's instructions. To eliminate genomic DNA contamination, RNA was treated with DNase I (TaKaRa) for 20 min. First‐strand cDNA was synthesized from total RNA using the TaKaRa RNA PCR kit. Real‐time PCRs were performed on an ABI 7500 real‐time PCR system using IQ5 multicolor real‐time PCR master mix (Toyobo). Each PCR (30 µl) contained 15 µl 2 × SuperReal Premix Plus, 0.25 µl each primer, and appropriate cDNA. PCR was performed under the following conditions: 95 °C for 15 minfollowed by 40 cycles of 95 °C for 10 s, 55–60 °C for 30 s, and 72 °C for 32 s. Melt curves were collected by the ABI 7500 system and followed by 95 °C for 15 s, 60 °C for 1 min, and then 95 °C for 30 s. *Ribosomal S16* (*S16*, At2g09990) and *elongation factor 1α* (*EF1α*, At5g60390) were used as internal references for all PCR analyses. The gene‐specific primers used for RT‐qPCR are listed in Table S1.

### 4.10 Drought stress and water loss

To apply drought stress, the protocol described by Seki et al. (2001) was used with minor modification. Seedlings were grown under well‐watered conditions for 4 weeks and subsequently deprived of water for 2 weeks. Next, the plants were rewatered for at least 3 days and photographed. For water‐loss assays, rosette leaves were collected from 5‐week‐old plants as test samples. The samples were weighed immediately on a piece of paper and placed on the laboratory bench (relative humidity 50%, 22–23 °C). The weight lost by each sample at preassigned time points (0, 1, 2, 3, 4, and 5 hr) was recorded.

### 4.11 Statistical analysis

For statistical analysis, one‐way analysis of variance (ANOVA) or two‐way ANOVA tests were performed with GraphPad prism 7.00 software.

## Supporting information


**FIGURE S1** *AtCERK1* mutation affects chitin‐induced stomatal closure. Mature leaf discs of Col‐0, *Atran1*, and *Atcerk1*plants with open stomata were exposed to either mock or 50 µg/ml chitin. Representative images from one of these experiments are shown. The stomatal apertures were measured after a 2‐hr incubation. At least 60 stomata were measured for each genotype per replication. Data were means ± *SE* of three independent experiments

**FIGURE S2** H_2_O_2_ triggers NO production. Epidermal fragments of Col‐0 were inoculated with DAF‐2DA. Fluorescence images and pixel intensities were recorded in the guard cells after addition of 100 mM H_2_O_2_. Data of fluorescence pixel intensities are displayed as means ± *SE*, *n* = 50 apertures per experiment. Means with different letters denote statistically significant differences among different treatments as determined by ANOVA (LSD test, *p *< .05)

**TABLE S1** Gene‐specific primers by RT‐PCR and RT‐qPCR

## Data Availability

The data that support the findings of this study are available in Arabidopsis eFP Browser at http://bar.utoronto.ca/efp_arabidopsis/cgi‐bin/efpWeb.cgi, reference number At5g20010. The GenBank accession number and Arabidopsis Genome Initiative (AGI) locus identifier of the *AtRAN1* gene are as follows: NM_122008.3 (GenBank), At5g20010 (AGI locus). Other data that support the findings of this study are available from the corresponding author upon reasonable request.
