# Supplementary material for: The Arabidopsis small G‐protein AtRAN1 is a positive regulator in chitin‐induced stomatal closure and disease resistance
Source: Mol Plant Pathol. 2020 Nov 15;22(1):92–107. doi: 10.1111/mpp.13010 (PMC7749754; doi:10.1111/mpp.13010)
Supplement: Supplementary file 1 — FIGURE S1 AtCERK1 mutation affects chitin‐induced stomatal closure. Mature leaf discs of Col‐0, Atran1, and Atcerk1plants with open stomata were exposed to either mock or 50 µg/ml chitin. Representative images from one of these experiments are shown. The stomatal apertures were measured after a 2‐hr incubation. At least 60 stomata were measured for each genotype per replication. Data were means ± SE of three independent experiments [file MPP-22-92-s001.doc]

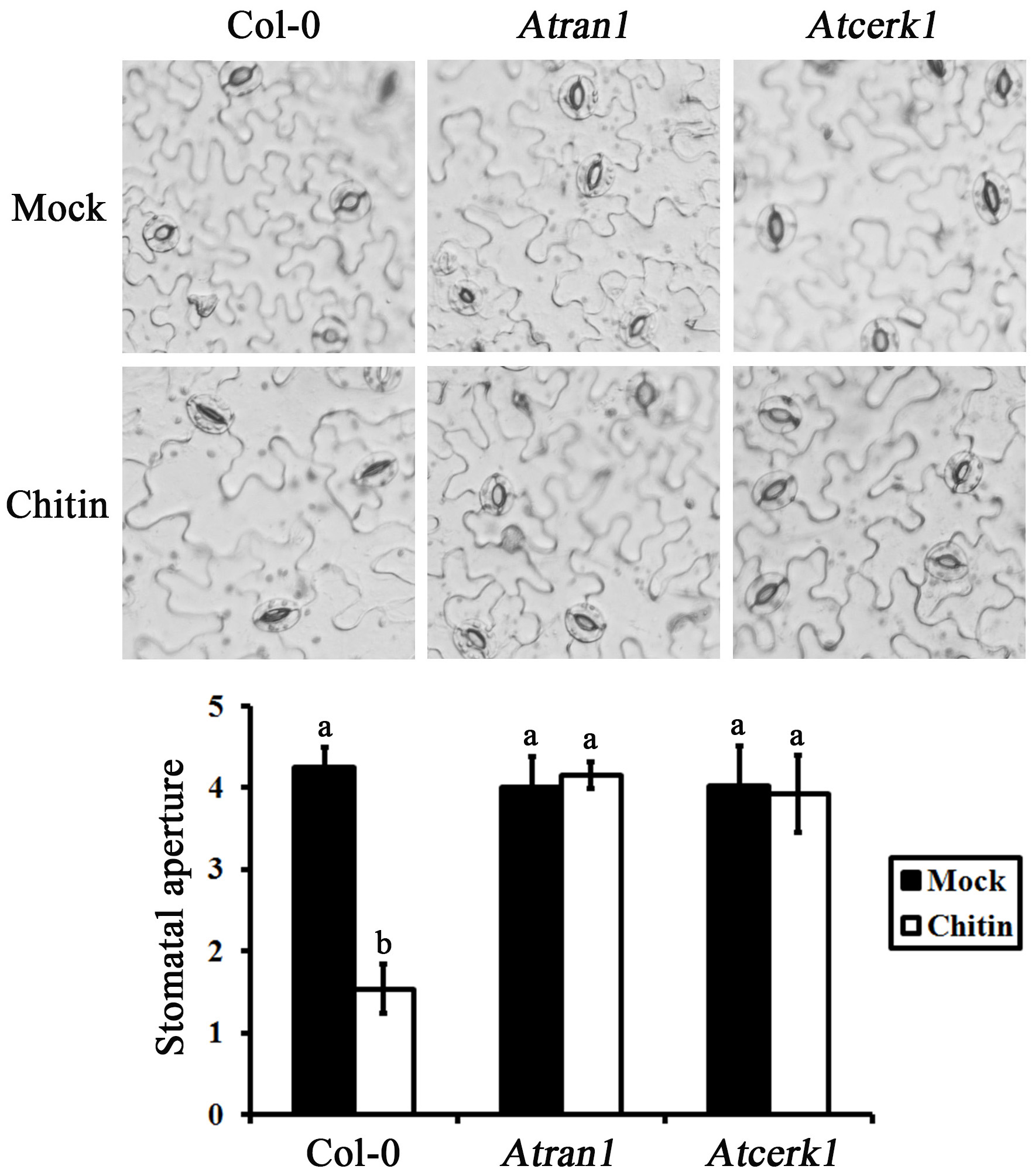


Figure S1 *Atcerk1* mutation affects chitin-induced stomatal closure.

Mature leaf discs of Col-0, *Atran1*, and *Atcerk1* plants with open stomata were exposed to either mock or 50 µg mL^-1^ chitin. Representative images from one of these experiments are shown. The stomatal apertures were measured after a 2-h incubation. At least 60 stomata were measured for each genotype per replication. Data were means ± SE of three independent experiments
