# Supplementary material for: The Arabidopsis small G‐protein AtRAN1 is a positive regulator in chitin‐induced stomatal closure and disease resistance
Source: Mol Plant Pathol. 2020 Nov 15;22(1):92–107. doi: 10.1111/mpp.13010 (PMC7749754; doi:10.1111/mpp.13010)
Supplement: Supplementary file 2 — FIGURE S2 H2O2 triggers NO production. Epidermal fragments of Col‐0 were inoculated with DAF‐2DA. Fluorescence images and pixel intensities were recorded in the guard cells after addition of 100 mM H2O2. Data of fluorescence pixel intensities are displayed as means ± SE, n = 50 apertures per experiment. Means with different letters denote statistically significant differences among different treatments as determined by ANOVA (LSD test, p < .05) [file MPP-22-92-s002.doc]

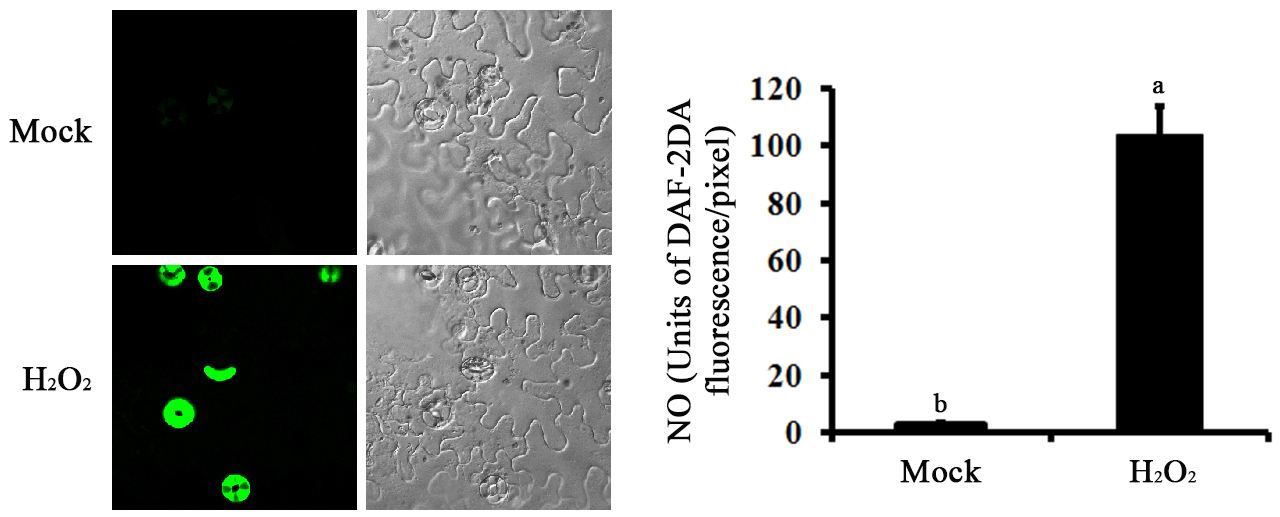


Figure S2 H_2_O_2_ triggers NO production.

Epidermal fragments of Col-0 were inoculated with DAF-2DA. Fluorescence images and pixel intensities were recorded in the guard cells after addition of 100 mM H_2_O_2_. Data of fluorescence pixel intensities are displayed as means ± SE, n=50 apertures per experiment. Means with different letters denote statistically significant differences among different treatments as determined by ANOVA (LSD test, *P*<0.05).
