# Supplementary material for: The Arabidopsis small G‐protein AtRAN1 is a positive regulator in chitin‐induced stomatal closure and disease resistance
Source: Mol Plant Pathol. 2020 Nov 15;22(1):92–107. doi: 10.1111/mpp.13010 (PMC7749754; doi:10.1111/mpp.13010)
Supplement: Supplementary file 3 — TABLE S1 Gene‐specific primers by RT‐PCR and RT‐qPCR [file MPP-22-92-s003.doc]

**Table S1 Gene-specific primers by RT-PCR and qRT-PCR**

| Primer name | Oligonucleotide sequence (5'-3') |
| --- | --- |
| AtRAN1promoter-F | ACGCGTCGACCTCAGTCTCATTAGCTTAAAG |
| AtRAN1promoter-R | CGCGGATCCAGCCATGTTTTCGCAATCG |
| AtRAN1-TL-F | CGGGGTACCATGGCTCTACCTAACCAGC |
| AtRAN1-TL-R | ACGCGTCGACCTCAAAGATATCATCATCG |
| AtRAN1-F | ATGGCTCTACCTAACCAGC |
| AtRAN1-R | TTACTCAAAGATATCATCATCG |
| AtRAN1-ResF | CGGGGTACCAATCGACATAGCTTTGTACCT |
| AtRAN1-ResR | CGCGGATCCATCACCTGGTAGCTAAAGAGT |
| RT-AtRAN1-F | CACAAACTGTGGCAAGATCCG |
| RT-AtRAN1-R | CACATCAACTTTGTTCCCACACAG |
| S16-F | GGCGACTCAACCAGCTACTGA |
| S16-R | CGGTAACTCTTCTGGTAACGA |
| EF1α-F | ACCACATGATTGAGAGGTCC |
| EF1α-R | GCATCTCAACAGACTTGACC |
| PR1-F | TcAcAcTcccgcTcAAccgccAAAA |
| PR1-R | ATcTcAcggAggcAcAAccAAgT |
| PDF1.2-F | cgggAAAATAAAcATTAAAAcAgAA |
| PDF1.2-R | ATATgcAgAcgcAccggcAATgg |
| PAL-F | cTTggcggAgAAAcAcTgAcgAT |
| PAL-R | AATATTccggcgTTcAAAAATcTgA |
| NIA-F | GCCATTATCCCACCATGAAC |
| NIA-R | GTTATGGCTCTCGTCTTCGT |
| RbohD-F | GAGAGCAGCCTCAACAACAC |
| RbohD-R | GTGGCAATGAACTTGAGACC |
| RbohF-F | CGGTGATCAAGAGTTCGTTG |
| RbohF-R | GCTCTTGTGAGAATTGTCGG |
| Lox-F | CAGACAGCTGAGTGTTCTTC |
| Lox-R | GTTCTGCTGGTAATGCTTGG |
| ERF1-F | CCTTCCGATCAAATCCGTAA |
| ERF1-R | GCAGCTTGATCGTAGGCTAA |
| RD29A-F | GACGGGATTTGACGGAGAACCAGAT |
| RD29A-R | TCCGGAGTAACCTAGCATTGAAGCA |
| KIN2-F | ACCAACAAGAATGCCTTCCA |
| KIN2-R | ACTGCCGCATCCGATATACT |
| AtCERK1-F | GTCGCGATTAGTAATTACGCG |
| AtCERK1-R | CGGAGTTAAAATTAACACCGGG |
